# School-Based Intervention to Improve Nutrition Knowledge and Lifestyle Awareness Among Adolescents: Results from an Italian Quasi-Experimental Study

**DOI:** 10.3390/nu18121861

**Published:** 2026-06-09

**Authors:** Gaia D’Antonio, Vincenza Sansone, Giovanna Paduano, Gabriella Di Giuseppe

**Affiliations:** Department of Experimental Medicine, University of Campania “Luigi Vanvitelli”, 80138 Naples, Italy; gaia.dantonio@unicampania.it (G.D.); vincenza.sansone@unicampania.it (V.S.); giovanna.paduano@unicampania.it (G.P.)

**Keywords:** adolescent, dietary habits, lifestyle behaviors, nutrition knowledge, school-based interventions

## Abstract

**Background/Objectives:** Adolescence is a critical period for the adoption of health-risk behaviors and the development of non-communicable diseases (NCDs). Schools represent a strategic setting for health promotion interventions; however, Italian studies simultaneously assessing NCD-prevention knowledge and lifestyle behaviors in the same adolescent population remain scarce. The study aimed to evaluate improvements in knowledge regarding nutrition and other lifestyle-related behaviors among Italian adolescents following a school-based educational intervention. Secondary objectives included describing lifestyle behaviors within the study population and exploring participants’ evaluation of the intervention. **Methods:** A quasi-experimental pre-post study was conducted between March and May 2025 in five lower secondary schools. A total of 410 adolescents aged 11–16 years were enrolled through a two-stage cluster sampling procedure. The intervention, lasting approximately two hours, was delivered by a trained nurse-researcher and addressed four health domains: nutrition, physical activity, screen exposure, and substance use. **Results:** Following the intervention, a measurable increase in overall knowledge scores (mean increase: +3.9 points) was observed, with 88.9% of participants showing improvement. The largest improvements were observed in nutrition-related knowledge and awareness of passive smoking harms. Despite these gains, unhealthy behaviors remained prevalent, including low adherence to physical activity recommendations (36.1%), suboptimal dietary quality (39.9%), and high screen exposure. A linear regression model identified five independent determinants of higher knowledge improvement: older age, female gender, higher screen exposure, having at least one employed parent, and lower pre-intervention test scores. The intervention was positively evaluated, with high levels of satisfaction, clarity, and perceived usefulness. **Conclusions:** Nevertheless, the persistent gap between knowledge and behavior underscores the need to integrate motivational and environmental components, gender-sensitive approaches, and longitudinal evaluations to foster sustainable, healthy choices and contribute to NCD prevention.

## 1. Introduction

Adolescence represents a critical developmental stage characterized by profound physical, psychological, and social changes that influence the adoption of health-related behaviors [1]. During this period, individuals progressively gain autonomy in lifestyle choices, including dietary habits, physical activity, sedentary behaviors, and substance use [2].

These choices, often established without full awareness of their long-term consequences, can track into adulthood and contribute to the development of non-communicable diseases (NCDs) [2,3]. NCDs, including cardiovascular diseases, cancer, diabetes, and chronic respiratory conditions, are the leading causes of mortality worldwide [4,5]. Sustainable Development Goal (SDG) 3.4 aims to reduce premature mortality from NCDs by one-third by 2030 [6]. Since less than five years remain, achieving this target requires comprehensive and integrated prevention strategies focused on modifiable behavioral risk factors, including tobacco use, harmful alcohol consumption, unhealthy diets, and physical inactivity [4,7,8]. In particular, in Southern Italy, unhealthy dietary behaviors and excess weight represent particularly pressing concerns among adolescents. Indeed, according to the 2022 Health Behaviours in School-aged Children (HBSC) Campania survey, overweight and obesity affect 31.7% of adolescents aged 11 to 15 years in the region, with poor dietary quality, physical inactivity, and high screen exposure clustering disproportionately among those from socioeconomically disadvantaged families [9]. Early prevention is therefore essential, requiring timely action on behavioral risk factors. Within this perspective, school-based health programmes are recognised as key components of multisectoral public health strategies aimed at supporting population-level progress [10].

Through their educational and social functions, schools can influence adolescents’ health literacy, behaviors, and well-being, while also indirectly affecting families and the wider community [11]. The school environment offers a unique opportunity to integrate health promotion into routine educational processes and to foster the translation of knowledge into everyday behaviors. This is particularly relevant for lifestyle behaviors, as adolescents’ choices are strongly influenced by contextual factors such as peer norms, environmental conditions, and the availability of healthy food options [12].

Evidence-based school interventions have demonstrated effectiveness in improving knowledge, changing attitudes, and, in some cases, positively influencing healthy behaviors among adolescents [13,14,15]. However, evidence on knowledge improvement following brief, multidomain educational interventions remains limited [16,17], and studies simultaneously assessing both health-related knowledge and lifestyle behaviors within the same adolescent population, particularly in the Italian context, are scarce. In this framework, the primary objective of the present study is to evaluate changes in adolescents’ knowledge regarding healthy and risky lifestyles following a school-based educational intervention. Secondary objectives are to assess participants’ perceptions of the intervention and to describe lifestyle behaviors within the study population.

## 2. Materials and Methods

### 2.1. Study Design, Setting, and Participants

A quasi-experimental pre-post study was conducted between 3 March and 31 May 2025 in the Campania region (Southern Italy), a populated area with approximately six million inhabitants and one of the highest youth population densities in the country [18]. Young adolescents, aged 11–16 years, attending lower secondary school were eligible for this study. The sample was selected through a two-stage cluster sampling procedure, structured to improve representativeness. In the first stage, five secondary schools were randomly selected from a list of regional public schools to form school clusters. In the second stage, classes were randomly selected from each school, and all students within the selected classes were invited to participate. The a priori sample size was estimated assuming a small standardized effect size (Cohen’s d = 0.30) for the expected improvement in adolescents’ knowledge of healthy and risky lifestyles. With a significance level of α = 0.05 (two-tailed) and a statistical power of 80%, the minimum required sample size was calculated to be 175 participants [19]. To account for the cluster sampling design (26 classes), a Design Effect of 1.44 was assumed, based on an average cluster size of 15.7 students per class and a conservative intraclass correlation coefficient (ICC) of 0.03. This adjusted the minimum required sample size to 252 participants. The final sample included 410 students, exceeding the minimum number required to ensure adequate statistical power. This study was approved by the Ethics Committee of the Teaching Hospital of the University of Campania “Luigi Vanvitelli” (approval number 0018199/i/01.07.2024).

### 2.2. Pilot Study

A pilot study involving 50 young adolescents was conducted to ensure the questionnaire was easy to understand and to answer. Pilot participants met the same eligibility criteria, received the same intervention, and completed identical pre- and post-test questionnaire procedures as the main sample. As no items required modification following the pilot study, participants were therefore included in the final analysis. The internal consistency reliability of the knowledge test was estimated using the Kuder-Richardson 20 (KR-20) formula, which is appropriate for dichotomous items. A KR-20 coefficient exceeding 0.70 is considered acceptable, above 0.80 indicates good reliability, and above 0.90 represents excellent reliability [20].

### 2.3. Data Collection and Educational Intervention

Before the educational intervention, school deans were contacted to arrange an informational meeting, during which a formal letter describing the study objectives and procedures was presented. Upon approval, each student received a sealed envelope to take home, containing a presentation of the study and the informed consent form. Participation was voluntary, and a written consent form was required from either a parent or a legal guardian. Anonymity and data protection were guaranteed, as clearly stated on the first page of the questionnaire.

The intervention was carried out in classrooms by trained nurse researchers who had completed a one-month methodological training focused on evidence-based recommendations for adolescent health promotion. To ensure consistency across schools, all researchers followed a standardized protocol and used the same slide-based presentation, which had been developed by the research team based on evidence sources and was not modified across sites. Implementation fidelity was supported through the use of a structured script accompanying the presentation. To minimize variability in delivery across sites and avoid potential contamination of knowledge measurements, students were not invited to raise questions during the educational session; any questions from participants were addressed informally by the researcher only after completion of the post-test. The intervention lasted approximately two hours. The session began with a slide-based presentation introducing the definition of NCDs [4,21], the specific features of adolescence [1,22], the importance of primary prevention, and the active role adolescents can play in promoting healthy lifestyles [23]. After the introductory section, students received a paper questionnaire structured in five sections: (1) sociodemographic and health-related characteristics; (2) lifestyle behavior; (3) pre-intervention knowledge test; (4) evaluation of the intervention; (5) post-intervention knowledge test. Students were first asked to complete the initial sections, collecting sociodemographic, health-related, and lifestyle information. The pre-intervention knowledge test was then administered by projecting each question on the classroom screen, while students recorded their answers on the questionnaire grid by selecting the option they considered correct. Researchers remained in the classroom during completion to verify that all questions had been answered and to minimize missing data. Following the pre-test, the main educational component of the session was delivered, presenting evidence-based recommendations on healthy eating [24,25], physical activity and screen exposure [26,27,28,29], smoking [30], and alcohol consumption [31]. At the end of the session, students evaluated the intervention, and the post-test was administered immediately after the intervention by using the same set of knowledge questions presented in a randomized order to reduce potential recall bias. Finally, students received a QR code linking to a digital educational brochure summarizing the key health messages of the intervention, including recommendations on balanced nutrition, daily physical activity, reduction in sedentary screen time, and the prevention of tobacco and alcohol use.

### 2.4. The Questionnaire and Measurement

A structured, self-administered questionnaire was developed to collect data on sociodemographic characteristics, lifestyle behaviors, pre-post intervention knowledge test, and evaluation of the intervention among adolescents. All items were grounded in current evidence-based recommendations from established national and international guidelines [24,25,26,27,28,29,30,31]. The instrument was reviewed internally by the research team to ensure content relevance, coverage of the four target health domains, and age-appropriate language.

#### 2.4.1. Sociodemographic and Health-Related Characteristics

Self-reported data were collected on participants’ age, gender, nationality, family structure (single-parent family, biparental family, or other), parental educational level and employment status, life satisfaction (LS), self-perceived health status, the presence of any NCD, and whether they knew someone affected by a NCD. LS was evaluated employing the Cantril ladder scale [32], which ranged from the lowest level 1, representing the worst possible life, to the highest level 10, indicating the best possible life. The self-perceived health status was evaluated using a ten-point Likert scale from 1 = poor to 10 = excellent [33].

#### 2.4.2. Lifestyle Measures

Lifestyle behaviors included dietary habits, physical activity, screen exposure, cigarette smoking, and alcohol consumption.

##### Dietary Habits

Dietary habits were assessed using questions about the number of meals per day, the frequency of breakfast, and the intake of fruit, vegetables, legumes, red meat, and sugar-sweetened beverages. These items were adapted from the Food Frequency Questionnaire module of the HBSC survey [34], with response categories ranging from “Never” to “Several times every day”. To evaluate dietary quality, a composite score was constructed using five indicators: eating breakfast on schooldays, daily consumption of fruit and vegetables, eating legumes at least twice per week, and limiting intake of carbonated sugary drinks to less than once per day. Each healthy behavior was assigned one point if present, resulting in a total score ranging from 0 to 5. The score was then dichotomised into poor dietary quality (<3 points) and good dietary quality (≥3 points), following the approach adopted by Nardone et al. [35].

##### Physical Activity and Screen Exposure

Physical activity was measured by asking students to report the average time spent in moderate to vigorous physical activity per day during a typical week. Responses were classified according to WHO guidelines, with adolescents engaging in ≥60 min per day considered as meeting the recommended level [26]. Screen exposure was defined as time spent passively watching screen-based entertainment (TV, computers, mobile devices), excluding active screen-based games requiring physical movement [36]. Participants reported the average time spent on screen-based entertainment during school days and weekends. Following international recommendations [28,29], screen exposure was dichotomised as meeting the recommendations only if they reported screen exposure below the 2 h daily threshold on both school days and weekends.

##### Substance Use

Tobacco and alcohol use were assessed using selected items from the European School Survey Project on Alcohol and Other Drugs (ESPAD) questionnaire, including measures of lifetime and past-year use [37].

#### 2.4.3. Evaluation of the Intervention

Students’ evaluation of the intervention was assessed with four items. The first three items measured satisfaction with the intervention, the clarity of the information provided, and the perceived usefulness of the session. Responses were rated on a five-point Likert scale ranging from 1 (not at all) to 5 (completely) and were dichotomised into high perception (score = 5) versus lower perception (scores 1–4). The fourth item assessed students’ willingness to participate in similar initiatives in the future and used a dichotomous response format (yes/no).

#### 2.4.4. Pre and Post-Intervention Knowledge Test

The knowledge assessment instrument was developed based on the content of the slide-based presentation delivered during the intervention [21,22,23,24,25,26,27,28]. The test evaluated knowledge across four health domains relevant to NCD prevention: nutrition, physical activity, screen exposure and sedentary behavior, and substance use. Questions were adapted in language and terminology to be age-appropriate for adolescents. The instrument consisted of 15 questions, yielding a total of 20 items. Specifically, the test included 11 single-response multiple-choice questions (each yielding 1 item), 2 questions with true/false statements (Q7 and Q15), each comprising 4 sub-statements scored independently (yielding 4 items each, for a total of 8 items), and 1 combined question (Q2–Q3) scored together as a single item. The test duration was 25 min. To support inclusive participation, a paper-based version of the test was made available to students identified by teachers as having attention difficulties to support their active participation.

### 2.5. Statistical Analysis

Data were analyzed using STATA version 18. Descriptive statistics, including frequencies and percentages, were used to summarize sociodemographic characteristics, lifestyle behaviors, intervention evaluation, and the proportion of correct answers for each knowledge item at both pre-test and post-test. Each knowledge item was scored dichotomously as correct or incorrect. The primary outcome was the overall knowledge score obtained by summing the number of correct responses on the 20-item test, assessed at two time points: before the intervention (pre-test) and immediately after the intervention (post-test). Scores were treated as continuous variables, and means and standard deviations were calculated. Only participants who completed both the pre-test and post-test were included in the comparative analysis, and incomplete paired data were treated as missing values. To assess whether the differences in scores met the assumption of normality required for parametric analyses, the Shapiro–Wilk test was used. The test indicated that the distribution of difference scores did not deviate significantly from normality (*p* > 0.05), supporting the use of parametric methods. A paired-samples *t*-test was therefore performed to compare mean knowledge scores between pre-test and post-test. Additionally, a Wilcoxon signed-rank test was performed as a non-parametric sensitivity analysis to further confirm the robustness of the findings. Item-level analyses (McNemar’s test for paired categorical data) were performed using all available paired responses, resulting in varying sample sizes across items. For each item, the number and percentage of correct responses at both time points were reported, along with responsiveness and *p*-value. Responsiveness was defined as the absolute percentage-point difference between post-test and pre-test proportions of correct responses (16); 95% confidence intervals were calculated using the paired proportion difference method, accounting for the paired nature of pre- and post-test observations. The overall paired knowledge score analysis was restricted to participants with complete data on all 20 items. Bivariate analyses were conducted using chi-square tests for categorical variables and independent samples *t*-tests for continuous variables to explore gender differences. A stepwise multivariate linear regression model was assessed to determine the predictors of knowledge score improvement following the intervention, defined as the difference between post-test and pre-test scores (continuous outcome). The values for variable entry and removal during the stepwise selection procedure were set at *p* = 0.2 and *p* = 0.4, respectively. The following independent variables were tested: age (continuous), gender (female = 0; male = 1), nationality (foreign = 0; Italian = 1), family structure (single-parent family/other = 0; biparental family = 1), having at least one parent with a university degree (no = 0; yes = 1), having at least one parent employed (no = 0; yes = 1), self-perceived life satisfaction (continuous), self-perceived health status (continuous), having at least one NCD (no = 0; yes = 1), knowing someone affected by a NCD (no = 0; yes = 1), dietary quality (poor = 0; good = 1), meeting WHO recommendation for physical activity (no = 0; yes = 1), meeting recommendations of screen exposure (no = 0; yes = 1), lifetime tobacco use (no = 0; yes = 1), lifetime alcohol use (no = 0; yes = 1), being satisfied with the educational intervention (no = 0; yes = 1), considering the information provided as clear (no = 0; yes = 1), perceiving the intervention as useful (no = 0; yes = 1), willingness to participate in similar interventions in the future (no = 0; yes = 1), and the pre-intervention test scores (continuous). All reported *p*-values are two-tailed, and statistical significance was set at *p* ≤ 0.05.

## 3. Results

### 3.1. Sample Characteristics, Lifestyle of the Adolescents, and Evaluation of the Intervention

#### 3.1.1. Sample Characteristics

Overall, a total of 452 students across 26 classes were identified as eligible, and 438 (96.9%) provided signed parental consent. On the day of the intervention, 410 students were present and completed the questionnaire, yielding a response rate of 93.6%. Attendance varied across schools, ranging from 91% to 98%. Adolescents reported a mean age of 13.4 years (range 11–16); slightly more than half (52.2%) were male, and only 3.4% were not Italian. Regarding family, 86.2% of adolescents lived with both parents, and 12.8% lived in a single-parent family. Furthermore, 53.1% had at least one parent with a university degree, and 98.5% reported at least one employed parent. When asked about self-perceived life satisfaction, adolescents reported an average of 7.6 (SD = 1.6) on a 10-point scale, and 44.1% rated their health as excellent. Additionally, 20.4% of participants suffered at least one NCD, whereas 55.5% declared knowing someone affected by a NCD. These socio-demographic and health-related characteristics of the sample are described in Table 1.

#### 3.1.2. Dietary Habits and Lifestyle Behaviors

Lifestyle behaviors were also explored, including dietary habits, physical activity, screen exposure, and substance use. Regarding the former, 24.7% of students reported eating five meals per day; 54.9% had breakfast on a daily basis, 35.3% consumed fruit, and 27.4% consumed vegetables every day, while 73.6% ate legumes at least twice per week.

Moreover, 44.3% of adolescents reported eating red meat once a week or less, while 30.4% reported limiting carbonated sugary drink intake to less than once per day. Overall, 39.9% of the sample were classified as having a good dietary quality.

With regard to physical activity and sedentary behavior, adolescents reported an average of 354 min of moderate-to-vigorous physical activity per week (range: 0–2340), although only 36.1% met the WHO recommendation of at least 60 min per day. Furthermore, screen exposure averaged 184.3 min (range 0–900) per day on schooldays and 225.5 min (range 0–900) on weekends. Based on international guidelines, one-third of the sample (33.8%) met the recommendation of less than two hours of screen time exposure per day. Concerning substance use, 14.8% of adolescents reported having smoked at least once in their lives, of whom 72.4% had smoked in the past year; while 34.5% of the sample reported lifetime alcohol use, with 77.3% consuming it in the past year. Table 2 displays the lifestyle characteristics of the sample by gender. Males were significantly more likely to meet the WHO recommendations of physical activity and to use alcohol during their lifetime (*p* < 0.001) compared with females.

#### 3.1.3. Evaluation of the Intervention

Students’ satisfaction with the intervention averaged 4.3 ± 0.7 points on a 5-point Likert scale, with 43.5% of adolescents reporting a high level. The clarity of the presented information received a mean score of 4.4 ± 0.8 points, with 56% of adolescents rating the clarity as high. Perceived usefulness of the intervention averaged 4.2 ± 0.9, with 46.3% indicating a high level of perceived utility.

Concerning future participation, 75.4% of students expressed willingness to participate in similar interventions. To gather additional insights, exploratory analyses were conducted to examine potential gender differences in intervention satisfaction and willingness to participate. Table 3 presents the evaluation of the intervention by gender. Females were significantly more likely to report high satisfaction with the intervention and a willingness to participate in similar interventions in the future (*p* = 0.007 and *p* = 0.002, respectively) compared to males.

### 3.2. Nutrition and Lifestyle Knowledge Test

**Figure 1:**
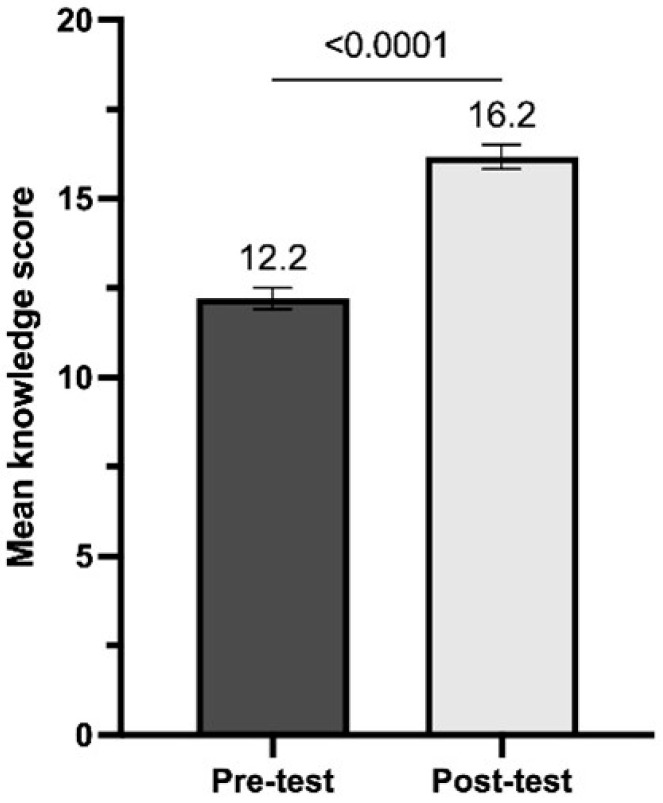
Change in mean knowledge scores from pre-test to post-test with 95% confidence intervals.

Of the 410 enrolled participants, 380 had complete paired pre- and post-test data and were included in the knowledge score analysis. Thirty participants were excluded due to missing responses on either the pre-test (n = 12) or the post-test (n = 18). The KR-20 reliability was estimated separately for pre-test and post-test responses. The pre-test yielded a KR-20 of 0.64, consistent with the heterogeneous baseline knowledge levels expected in a general adolescent population before instruction. The post-test yielded a KR-20 of 0.83, indicating good internal consistency, though this value may partly reflect short-term learning effects given the immediate post-intervention administration. In the pre-test (N = 380), the average score of correctly marked items was 12.2 ± 3.0 [2,3,4,5,6,7,8,9,10,11,12,13,14,15,16,17,18,19] (95% CI: 11.9–12.5), while in the post-test (N = 380), the average score increased to 16.2 ± 3.3 [3,4,5,6,7,8,9,10,11,12,13,14,15,16,17,18,19,20] (95% CI: 15.8–16.5). The mean difference between post- and pre-test scores was 3.9 ± 2.8 (95% CI: 3.7–4.3), corresponding to a 19.5% increase in the percentage of correctly marked items. The improvement, reported by 88.9% of participants, was statistically significant (*t*-test (379) = −27.0722, *p* < 0.001). A Wilcoxon signed-rank test performed as a sensitivity analysis confirmed the significance of the improvement (z = −16.025, *p* < 0.001, r = 0.82). This improvement in mean knowledge scores is illustrated in Figure 1.

Analysis of individual knowledge items at baseline revealed varying levels of understanding across different health domains. After the intervention, 17 of the 20 analysed items showed significant improvements in correct answers as assessed by the McNemar test, with detailed results reported in Table 4.

Exploring gender differences in knowledge score, females demonstrated significantly higher pre-test scores than males (mean: 12.7 ± 3 and 11.8 ± 3, respectively; *p* = 0.003) and significantly higher post-test scores (mean: 17.1 ± 2.6 and 15.3 ± 3.7, respectively; *p* < 0.001). At baseline, the lowest levels of knowledge were observed for the recommended number of daily meals for adolescents (36.1%) and the recommended portions of fruit and vegetables (47.7%). The greatest gains were recorded for these two nutrition items, with responsiveness values of 43.8% and 40.7%, respectively (*p* < 0.001).

Concerning physical activity, more than half knew the recommended daily duration at baseline (56.7%) and improved significantly after the intervention (responsiveness 23.1%, *p* < 0.001). In terms of screen exposure and sedentary behavior, awareness of the link between prolonged screen time and increased risk of heart disease was particularly low at baseline (27.4%) and improved significantly after the intervention (responsiveness 22.4%, *p* < 0.001). Regarding substance use, awareness of the health harms of passive smoking was low at baseline (25.7%) and showed the most marked improvement in this domain (responsiveness 41%, *p* < 0.001), while awareness of the health consequences of alcohol consumption also improved significantly (responsiveness 24.1%, *p* < 0.001).

### 3.3. Multivariate Analysis

The stepwise multivariate linear regression model showed that older adolescents, females, participants with higher screen exposure, those with at least one employed parent, and those with lower pre-intervention test scores were associated with a greater improvement in knowledge scores following the intervention (Table 5).

## 4. Discussion

This study evaluated changes in adolescents’ lifestyle-related knowledge following a school-based intervention and described their health behaviors. This is, to our knowledge, the first Italian pre–post study assessing NCD-prevention knowledge across multiple domains. Nevertheless, lifestyle behaviors were assessed at baseline only and were not reassessed after the intervention. Although the clustering effect was considered during the sample size calculation, it was not explicitly accounted for in the statistical analyses. This may have resulted in an underestimation of standard errors and, consequently, a potential overestimation of the statistical significance of the observed associations. Therefore, the results should be interpreted with caution.

At baseline, knowledge of dietary recommendations was suboptimal, with fewer than half of participants correctly identifying fruit and vegetable intake guidelines. Indeed, adequate intake during adolescence has been associated with improved cognitive performance, long-term weight control, and lower chances of developing NCDs in adulthood [38]. In contrast, awareness regarding breakfast frequency was notably higher (84.3%), suggesting that some nutrition-related messages may be more effectively internalized, likely due to their integration into daily family routines. Knowledge was higher after the intervention. However, dietary behaviors remained suboptimal, with low daily consumption of fruit and vegetables. This pattern aligns with European data from the HBSC study, showing that fewer than 40% of adolescents consume fruit or vegetables daily and that more than half do not consume them every day [39].

These findings confirm that poor dietary habits are widespread across European adolescents and not limited to the Italian context. These findings are consistent with national data. Regular breakfast consumption (54.9%) was more aligned with European averages reported in the HBSC 2021–2022 survey, where 51% of adolescents consumed breakfast daily, with considerable variation across countries [39]. The relatively favourable breakfast pattern observed in Italy may be influenced by enduring family routines and parental involvement in morning meals. A clear gap between baseline knowledge and behavior emerged at the time of enrolment. While awareness of certain nutritional recommendations was relatively high, this did not consistently translate into healthy dietary practices. This discrepancy underscores that baseline knowledge, while necessary, is insufficient on its own to promote healthy dietary choices, suggesting that environmental and social factors play a key role beyond knowledge alone [40].

At baseline, knowledge of physical activity recommendations was moderate. Knowledge was higher after the intervention. However, only a minority met recommended activity levels. This finding is consistent with European and global evidence indicating that a large proportion of adolescents fail to meet physical activity recommendations and that inactivity remains highly prevalent [39,41]. Results confirm the well-known knowledge–behavior gap in adolescents, highlighting a discrepancy between knowledge of physical activity guidelines and reported behavioral adherence [42,43]. Males were more likely to meet recommendations than females. This gender disparity is consistent with global evidence that mirrors patterns described in European surveillance systems, where girls consistently report lower physical activity levels than boys [44,45]. The gender gap in physical activity reflects an evidence-based phenomenon that is shaped by socio-cultural norms, differential access to sport facilities, peer dynamics, and individual motivational factors [46].

Baseline awareness of screen-related risks was variable. Knowledge was higher after the intervention. This is relevant given the established association between screen time and cardiometabolic risk [47,48]. This limited awareness becomes even more concerning when considered alongside the behavioral data. Behavioral data showed that most participants exceeded recommended limits. Following European trends, screen-based entertainment use has increased dramatically among adolescents, with insufficient awareness of associated health consequences, including disrupted sleep patterns, reduced physical activity, and increased cardiovascular risk [36,49]. These findings support integrating screen-health literacy into school curricula.

Knowledge of traditional tobacco risks was relatively high at baseline. Awareness of second-hand smoke risks was low but improved markedly. Similarly, awareness of e-cigarette risks was moderate and was higher post-intervention, which is particularly relevant in the European context, where e-cigarette use among adolescents has risen substantially [50].

Recent WHO data indicate that e-cigarette use among adolescents aged 15–16 years averaged 22% across the WHO European Region in 2024, ranging from 6.4% in Portugal to 36% in Poland, and exceeding 30% in five countries (Croatia, Czechia, Hungary, Serbia, and Poland) [51]. Adolescents may underestimate vaping-related risks [52,53]. Most participants recognized that alcohol is unsafe during adolescence, although knowledge gaps remained. Of greater concern, however, is the coexistence of high rates of lifetime alcohol use (34.5%) with incomplete risk awareness, particularly among males who reported significantly higher lifetime consumption than females, although the analysis is exploratory. In addition, lifetime alcohol use remains highly prevalent in Europe, with approximately three-quarters (73%) of adolescents reporting experience with alcohol [54]. These findings highlight the need for early, comprehensive prevention strategies.

The regression model identified five independent determinants of knowledge score improvement following the school-based intervention. Older adolescents demonstrated greater gains, consistent with evidence that health literacy levels vary significantly with age, with younger students consistently reporting lower health literacy than their older peers [55]. Participants with lower pre-intervention scores improved the most, a pattern observed in comparable school-based interventions where higher baseline knowledge predicted higher post-intervention scores, suggesting that adolescents starting from a more limited knowledge base benefit most from structured educational inputs [56]. Female gender independently predicted higher gains, aligning with the literature suggesting that girls often demonstrate stronger engagement with health-promotion programs and higher health literacy responsiveness compared to their male peers in school settings [57,58]. Having at least one employed parent was associated with larger improvements, which is in line with previous studies describing how parents’ employment is a predictor of academic achievement [59,60]. Finally, higher screen exposure predicted knowledge improvement, potentially because adolescents who are highly familiar with digital and multimedia environments are more primed to engage effectively with visually structured, slide-based educational delivery; as documented in a study evaluating an interactive multimedia nutrition education program, where pre-existing exposure to digital formats supported engagement with and retention of structured educational content [61].

Overall, after the intervention, a modest increase in health-related knowledge scores was observed, although causal inference is limited by the single-arm pre-post design. Most items showed significant improvement. The three items that did not improve significantly were: awareness that screen time does not improve attention (Q7a), cigarette smoking harms (Q9), and the inability of sugary drinks to replace fruit (Q15b). All had baseline performance exceeding 75% (78.4%, 78.1%, and 86.6%, respectively), suggesting that a ceiling effect may have limited the potential for measurable gains in these domains. These findings are consistent with previous school-based interventions [15]. Similarly, a pre-post study conducted among middle and high school students demonstrated that a brief educational intervention was sufficient to produce a significant increase in cancer literacy scores, from 50.2% to 77.1% of correct responses immediately after the intervention [16]. Nevertheless, despite the observed increased knowledge, it is important to underline that the post-test was administered immediately after the intervention using the same questions, which may have introduced a short-term recall effect and partially overestimated the observed improvement in scores.

Beyond knowledge outcomes, the intervention was well received by participants, who reported high levels of satisfaction, clarity, and perceived usefulness, with approximately three-quarters (75.4%) expressing willingness to engage in similar initiatives in the future. Females showed significantly greater satisfaction and openness to future participation compared to males, suggesting a need to better explore the role of gender in the development of health literacy. These findings suggest that future studies could explore whether gender-tailored or more interactive strategies improve engagement among boys.

### Limitations

Several methodological considerations should be acknowledged. First, the absence of a control group in this single-arm pre-post study limits causal inference. Improvements in knowledge cannot be definitively attributed to the intervention alone, as external factors or test–retest effects may have contributed to the observed changes. Nevertheless, this quasi-experimental design represents a pragmatic and widely adopted approach in school-based health promotion research, where randomized controlled trials are often constrained by ethical, organizational, and logistical barriers. Second, knowledge was assessed immediately after the educational session, obtaining only short-term knowledge acquisition. The absence of delayed follow-up measurements limits the conclusion regarding knowledge retention, skill consolidation, and behavioral change over time. Longitudinal designs incorporating follow-up assessments at three, six, or twelve months would provide stronger evidence regarding sustained effectiveness. Moreover, the knowledge test was developed specifically for this intervention; while it underwent a pilot phase to ensure comprehension, its psychometric validity remains limited. Third, the regional nature of the sample may limit the generalizability of findings to other geographic areas or educational contexts. Fourth, self-reported data are susceptible to social desirability and recall bias. Fifth, voluntary participation requiring parental consent may have generated self-selection bias, potentially overrepresenting students with greater baseline interest in health-related topics and thereby inflating the estimated intervention effect. Sixth, lifestyle behaviors were assessed at baseline only and were not reassessed after the intervention; therefore, the study cannot evaluate behavioral change, but only immediate knowledge change. Seventh, the statistical analysis did not formally account for the clustering effect at the school or classroom level. Because students within the same class or school are exposed to shared environments, teaching practices, and peer influences, their responses may be correlated, violating the assumption of independence of observations. This limitation is particularly important in school-based research and should be carefully considered when interpreting the findings. Lastly, several items were correctly answered by >75%, suggesting a ceiling effect, which may have constrained the measurable range of knowledge gain. Indeed, the knowledge assessment instrument was developed specifically for this study and, while grounded in evidence-based guidelines and piloted for comprehension, did not undergo formal psychometric validation. Future research incorporating controlled designs, extended follow-up periods, and validated standardized tools should adopt analytical approaches that explicitly account for data clustering, and objective behavioral indicators would strengthen the evidence on the effectiveness of school-based interventions in adolescents.

## 5. Conclusions

School-based interventions may represent an effective strategy for improving adolescents’ immediate knowledge of NCD risk factors, confirming the central role of schools as key settings for health promotion. Since high knowledge does not consistently translate into healthy behavior at baseline, further studies should provide a follow-up to explore if the intervention has an impact on long-term behaviors. This gap, also observed at the European level, highlights the need to move beyond purely informational approaches. Educational systems should therefore be more strategically leveraged not only to disseminate evidence-based health guidance but also to provide supportive environments that make healthy choices accessible, feasible, and sustainable. Effective interventions should integrate educational, motivational, and environmental components, adopt gender-sensitive approaches, and include long-term and multisectoral strategies. Engaging adolescents early and more systematically in health education is essential to establish behavioral patterns that persist into adulthood and, within a broader system-level prevention framework, contribute to reducing the long-term burden of NCDs at the population level.

## Figures and Tables

**Table 1:** Sociodemographic and health-related characteristics of the sample.

Characteristics	Total (N:410)	
	**N**	**%**
Age	13.4 ± 1.4 (11–16) *	
Gender (408) ^a^		
Female	195	47.8
Male	213	52.2
Nationality (409) ^a^		
Other	14	3.4
Italian	395	96.6
Family structure		
Single-parent family	52	12.8
Biparental family	349	86.2
Alternative family structure	4	1
Parents’ Educational level (407) ^a^		
Other	191	46.9
At least one parent with a university degree	216	53.1
Parents’ employment status (398) ^a^		
Other	6	1.5
At least one parent employed	392	98.5
Self-perceived life satisfaction (405) ^a^	7.6 ± 1.6 (1–10) *	
Self-perceived health status (408) ^a^	7.9 ± 1.8 (1–10) *	
Poor	5	1.2
Fair	14	3.4
Good	57	14
Very good	152	37.3
Excellent	180	44.1
Presence of any NCDs (398) ^a^		
No	317	79.6
At least one	81	20.4
Knowing someone affected by an NCD (398) ^a^		
No	177	44.5
Yes	221	55.5

* Mean ± Standard deviation (range); ^a^ Number of respondents for each item.

**Table 2:** Lifestyle characteristics of the sample by gender.

Lifestyle Characteristics	Tota (N:410)		Female	Male
	N	%	N (%) *	N (%) *
Dietary quality (389) ^a^				
Poor	234	60.1	118 (50.4)	116 (49.6)
Good	155	39.9	67 (43.5)	87 (56.5)
			χ^2^ (1) = 1.78, *p* = 0.182	
Meeting WHO recommendation for physical activity (402) ^a^				
No	257	63.9	143 (56.1)	112 (43.9)
Yes	145	36.1	51 (35.2)	94 (64.8)
			χ^2^ (1) = 16.17, *p* < 0.001	
Meeting recommendations of screen exposure (379) ^a^				
No	251	66.2	112 (45)	137 (55)
Yes	128	33.8	69 (53.9)	59 (46.1)
			χ^2^ (1) = 2.70, *p* = 0.100	
Lifetime tobacco use (398) ^a^				
No	339	85.2	166 (49.1)	172 (50.9)
Yes	59	14.8	21 (36.2)	37 (63.8)
			χ^2^ (1) = 3.31, *p* = 0.069	
Lifetime alcohol use (394) ^a^				
No	258	65.5	139 (54.1)	118 (45.9)
Yes	136	34.5	46 (34.1)	89 (65.9)
			χ^2^ (1) = 14.22, *p* < 0.001	

^a^ Number of respondents for each item; * Gender-stratified analyses were conducted among participants with available gender data. The sum of female and male counts may not equal the overall total due to missing data.

**Table 3:** Evaluation of the intervention of the sample by gender.

Evaluation of the Intervention	Total (N:410)		Female	Male
	N	%	N (%) °	N (%) °
High satisfaction with the intervention (391) ^a^	4.3 ± 0.7 (1–5) *			
No	221	56.5	96 (43.4)	125 (56.6)
Yes	170	43.5	96 (57.1)	72 (42.9)
			χ^2^ (1) = 7.17, *p* = 0.007	
High clarity of information presented (391) ^a^	4.4 ± 0.8 (1–5) *			
No	172	44	79 (45.9)	93 (54.1)
Yes	219	56	113 (52.1)	104 (47.9)
			χ^2^ (1) = 1.45, *p* = 0.229	
High perceived usefulness of the intervention (389) ^a^	4.2 ± 0.9 (1–5) *			
No	209	53.7	95 (45.7)	113 (54.3)
Yes	180	46.3	96 (53.3)	84 (46.7)
			χ^2^ (1) = 2.27, *p* = 0.132	
Willingness to participate in similar interventions in the future				
No	101	24.6	35 (34.6)	66 (65.4)
Yes	309	75.4	160 (52.1)	147 (47.9)
			χ^2^ (1) = 9.29, *p* = 0.002	

^a^ Number of respondents for each item; * Mean ± Standard deviation (range); ° Gender-stratified analyses were conducted among participants with available gender data. The sum of female and male counts may not equal the overall total due to missing data.

**Table 4:** Knowledge question items with correct answer, pre and post-test scores, and responsiveness.

	Number of Correct Answers (%)			McNemar Test		
Knowledge Question	N *	Pre-Test	Post-Test	Responsiveness (%)	95% CI (%)	*p*-Value
**Q1.** How many meals should an adolescent eat each day?	404	146 (36.1)	323 (79.9)	43.8%	38.4–49.3	<0.001
**Q2–Q3.** How many portions of fruit and vegetables should an adolescent eat each day?	405	193 (47.7)	358 (88.4)	40.7%	35.3–46.1	<0.001
**Q4.** How many times per week should an adolescent eat breakfast?	407	343 (84.3)	371 (91.2)	6.9%	2.5–11.2	0.001
**Q5.** What is a benefit of regular physical activity?	405	239 (59)	312 (77)	18%	12.3–23.8	<0.001
**Q6.** How many minutes of moderate-to-vigorous physical activity are recommended daily for adolescents?	406	230 (56.7)	324 (79.8)	23.1%	17.4–28.9	<0.001
**Q7.** True or False questions on spending too much time in front of a screen						
It improves attention	403	316 (78.4)	321 (79.6)	1.2%	−3.9–6.4	0.691
It increases the risk of obesity	401	224 (55.9)	334 (83.3)	27.4%	22.0–32.9	<0.001
It increases the risk of heart disease	398	109 (27.4)	198 (49.8)	22.4%	16.7–28	<0.001
It improves reflexes	402	219 (54.5)	328 (81.6)	27.1%	21.6–32.6	<0.001
**Q8.** How many hours per day should an adolescent spend in sedentary activities?	402	233 (58)	321 (79.9)	21.9%	16.6–27.2	<0.001
**Q9.** What harm(s) does cigarette smoking cause to health?	406	317 (78.1)	332 (81.8)	3.7%	−0.8–8.2	0.11
**Q10.** Which substance found in cigarette smoke causes addiction?	407	346 (85)	368 (90.4)	5.4%	0.9–9.8	0.015
**Q11.** What harm(s) does passive smoking cause to health?	405	104 (25.7)	270 (66.7)	41%	34.9–47	<0.001
**Q12.** Which of the following statements about e-cigarettes is true?	407	234 (57.5)	331 (81.3)	23.8%	18.3–29.4	<0.001
**Q13.** What harm(s) does alcohol consumption cause to health?	398	208 (52.3)	304 (76.4)	24.1%	18.7–29.5	<0.001
**Q14.** What is the maximum recommended alcohol consumption for adolescents?	406	295 (72.7)	349 (86)	13.3%	8.6–18	<0.001
**Q15.** True or False questions on carbonated sugary drinks						
They can improve sports performance	404	229 (56.7)	294 (72.8)	16.1%	10.9–21.3	<0.001
They can replace fruit	403	349 (86.6)	352 (87.3)	0.7%	−3.5–5	0.809
They help with concentration and studying	403	195 (48.4)	286 (71)	22.6%	17–28.2	<0.001
They can lead to weight gain	402	358 (89)	378 (94)	5%	1.4–8.6	0.006

* Number of each item may not add up to the total number of study populations due to missing values.

**Table 5:** Results of the multivariate linear regression models.

Variable *	β	SE	t	*p*
Model. Knowledge score improvement F (8, 283) = 13.92; R^2^ = 28.2%; adjusted R^2^ = 26.2%; *p* < 0.001				
Female	−1.45	0.31	−4.69	<0.001
Pre-intervention test scores	−0.42	0.05	−8.56	<0.001
Do not meet the recommendations of screen exposure	−1.18	0.31	−3.78	<0.001
Having at least one parent employed	3.09	1.10	2.80	0.006
Older	0.24	0.11	2.15	0.032
Knowing someone affected by an NCD	0.44	0.29	1.51	0.132
Having a biparental family	0.58	0.41	1.40	0.164
Italian	1.06	0.83	1.29	0.199

* The following variables were deleted during the stepwise procedure: dietary quality, having at least one parent with a university degree, meeting WHO recommendation for physical activity, self-perceived life satisfaction, willingness to participate in similar interventions in the future, considering the information provided as clear, lifetime tobacco use, lifetime alcohol use, having at least one NCD, self-perceived health status, perceiving the intervention as useful, being satisfied with the educational intervention.

## Data Availability

The data presented in this study are available upon request from the corresponding author due to privacy reasons.

## Abbreviations

The following abbreviations are used in this manuscript:

ESPAD	European School Survey Project on Alcohol and Other Drugs
HBSC	Health Behaviours in School-aged Children
ICC	Intraclass Correlation Coefficient
LS	Life Satisfaction
KR-20	Kuder-Richardson 20
NCDs	Non-Communicable Diseases
WHO	World Health Organization
